# Serum levels of PIICP, PIIANP, and PIIBNP are decreased in patients with an endemic osteochondropathy, Kashin-Beck disease

**DOI:** 10.1186/s13018-018-0840-z

**Published:** 2018-05-29

**Authors:** Wei Lian, Hui Liu, Li Yan Sun, Yun Qi Liu, Si Lu Cui, Yue Wang, Quan Quan Song, Qing Deng, Shao Ping Wang, Yan Hong Cao, Xue Ying Zhang, Yuan Yuan Jiang, Hong Yan Lv, Li Bin Duan, Jun Yu

**Affiliations:** 1 Institute for Kashin-Beck Disease Control and Prevention, Chinese Center for Disease Control and Prevention, Harbin Medical University, Harbin, China; 2 Key Laboratory of Etiology and Epidemiology, National Health and Family Planning Commission, Harbin, 23618504 China; 3 Jilin Institute of Endemic Disease Prevention Second, Jilin, China

**Keywords:** Kashin-Beck disease, Type II collagen, PIICP, PIIANP, PIIBNP, Biomarkers

## Abstract

**Background:**

Kashin-Beck disease (KBD) is an endemic, chronic, degenerative osteoarthropathy. KBD is usually diagnosed by using X-ray image and clinical symptoms, lacking of serological biomarkers. The serum level of PIICP, PIIANP, and PIIBNP can specifically reflect the damage of the cartilage. So, in this study, the serum levels of PIICP, PIIANP, and PIIBNP were detected in order to determine whether they can be used as potential biomarkers for the diagnosis of KBD.

**Method:**

Using a status survey, the survey sites were selected in the KBD historical endemic areas and non-endemic areas in Jilin and Heilongjiang provinces. All local residents have undergone clinical examination, X-ray examination of the hands and knees, and questionnaire survey. A total of 554 people were surveyed, and 184 residents who are eligible for inclusion criteria were selected as our subjects. Fifty-six cases were diagnosed as KBD and 63 individuals were included as internal control and 65 subjects were included as external control. And blood samples of surveyed subjects were collected, and the serum was separated to detect the levels of PIICP, PIIANP, and PIIBNP by ELISA. Statistical analysis was performed using the SPSS software.

**Results:**

There were no statistically significant differences in age and sex among the three groups. The Kruskal-Wallis *H* test showed that the serum levels of PIICP, PIIANP, and PIIBNP were significantly different among the three groups. Multiple comparisons using Dunnett’s T3 test revealed that serum levels of PIICP, PIIANP, and PIIBNP were significantly lower in KBD patients than in internal and external control. However, there was no significant difference between the internal and external control.

**Conclusions:**

The results preliminarily indicated that the levels of PIICP, PIIANP, and PIIBNP in serum could reflect the abnormal synthesis of type II collagen in KBD patients and suggested that these indicators might be used as potential biomarkers for the diagnosis of KBD.

## Background

Kashin-Beck disease (KBD) is an endemic, chronic, and deformative osteoarthropathy that mainly occurs in children aged 5–15 years old [1], and it was named such by the international medical community [2]. The disease is known for the formation of multi-joint hyperplasia bone changes [3]. KBD has a high prevalence in the broad diagonal belt from northern-east to southern-west in China. And the disease can also be found in Siberia and a few areas in North Korea. Most of the endemic areas are located in the cold and arid regions of warm and humid areas [4]. Although KBD has been studied over 160 years, the etiology is still unclear. There are three main etiological hypotheses, namely biogeochemical theory (mainly selenium deficiency), fusarium toxin (mainly T-2 toxin) poisoning theory of food, and drinking water organism poisoning theory.

Articular cartilage has an important role in cushioning the joints of skeleton, and it is mainly composed of type II collagen and various proteoglycans including aggrecan. Changes in the quality and quantity of type II collagen both are the direct cause of loss of their normal biomechanical properties [5], and these changes are closely related to KBD [6]. Type II collagen is synthesized by chondrocytes. The immature protein contains three extra domains: a signal peptide and N-terminal and C-terminal propeptide domains when propeptide regions upon cleavage allow mature collagen molecules to be incorporated into the extracellular matrix [7]. As C-terminal propeptide (PIICP) are released only during synthesis of the new molecules, its production is known to reflect the rate of type II collagen synthesis and the cartilage metabolism [8–10]. IIA and IIB procollagen N-terminal propeptide (PIIANP, PIIBNP) is the N-terminal non-helical structure cleaved when type II collagen precursor forms type II collagen, which also reflects the anabolic status of articular cartilage [11, 12]. Now, with the rapid development of modern molecular biology technology, the study of products in the process of type II collagen synthesis has also advanced.

The diagnosis of KBD usually adopts the X-ray examination (mainly hand images); the deformation of the interphalangeal joint is the basic characteristic of KBD. The different degrees of shortening of the fingers, limbs, and body are the main gist in diagnosing and classifying the severity of Kashin-Beck disease [13]. And when the disease could be diagnosed, the cartilage was already damaged badly and clinical signs and symptoms appear. The currently available treatments for KBD are limited to nonspecific interventions, pharmacologic management of the symptoms, and surgical treatment for severe adult KBD [14–16]. Non-steroidal anti-inflammatory drugs and analgesics are heavily used to alleviate patients’ pain because these agents are inexpensive. However, these agents are accompanied by a high risk of adverse events [14]. Therefore, the effective early diagnosis of KBD before the cartilage was damaged badly is an important step toward improving the management of this disease.

As mentioned above, measuring the level of some molecules related to type II collagen synthesis in the serum could reflect the degree of cartilage metabolism, and it might be helpful to understand the pathogenesis of KBD. Therefore, the aim of this study was to identify changes in the serum levels of PIICP, PIIANP, and PIIBNP in KBD patients and explore the possibility of these indicators as diagnostic biomarkers of KBD.

## Methods

### The selection of investigation sites

#### Selection criteria of investigate villages

Selection principle of KBD endemic villages are the following: ① there is history of being a KBD endemic area; ② KBD monitoring data is complete; ③ X-ray positive detection rate of children < 3%; and ④ the population of residents over age 40 is more than 100 people.

Selection criteria of non-KBD villages are the following: ① there is no history of being a KBD endemic area; ② the population of residents over age 40 is more than 100 people; and ③ eating habits and economic level of villagers are similar to KBD villages.

According to our selection criteria, the survey sites were selected in Jilin and Heilongjiang provinces. They are Dongxia and Zhoujia villages (Songyuan City of Jilin Province), Hanxia and Youhao villages (Jiaohe City of Jilin Province), and Yushu cha and Puban shi villages (Tonghua City of Jilin Province) in KBD endemic areas and Sanjing Village (Songyuan City of Jilin Province), Fuqiang Village (Jiaohe City of Jilin Province), and Heigang Village (Qiqihaer City of Heilongjiang Province) in non-KBD areas.

### Selection of study subject

Clinical examination, radiologic examinations, and questionnaire survey were performed for the adults over 40 years of age who were living in the abovementioned villages. After the examinations and questionnaire survey, the eligible individuals were selected. The inclusion criteria of KBD patients (KBD was diagnosed in accordance with the “Chinese Diagnosis of Kashin-Beck Disease” standard (WS/T 207-2010) for the diagnosis of Kashin-Beck disease) are the following: (1) the subject lived in KBD endemic area in childhood and adolescence for more than 6 months; (2) there are multiple, symmetric finger joint thickening or short digit (toe) or other symptoms of KBD in subjects; and (3) excluding other bone and joint diseases. The inclusion criteria of the control are the following: ① adults without KBD, OA, and other bone or joint diseases, such as joint inflammation, metabolic bone diseases, neoplasia, osteoporosis, or osteomalacia; ② no history of traumatic knee disease and liver and/or kidney diseases; ③ not overweight, BMI ≤ 30; and ④ not receiving hormones or any other medication that affects bone metabolism.

### Detection equipment and methods

#### Detection equipment

The detection equipment used for a high-frequency portable digital medical diagnostic X-ray image DR system were the following: (1) X-ray generator, (2) flat panel detectors, and (3) portable video workstation. The computer model is a Lenovo computer Y450.

#### Detection method

The radiologic examination sites are the metacarpophalangeal joint and knee joint.

### Quality control

Professionals firstly read the images based on the Chinese Diagnosis of Kashin-Beck Disease. In order to ensure the quality of data, all abnormal X-ray images were examined again by three experts. The experts provide their own evaluations to our team, and we finalized the diagnoses of the patients according to the views of these experts.

### Serum samples and laboratory measures

Five-milliliter samples of peripheral venous blood of the upper arm were collected in the morning from each patient and control participant. The blood samples were left at room temperature for 1 h and then were centrifuged at 3000 rpm/min for 20 min to separate the serum. The serum was dispensed into 50 μl aliquots into micro centrifuge tubes and then was stored at − 80 °C until assay.

Assays were carried out to determine serum levels of PIICP, PIIANP, and PIIBNP by a sandwich enzyme-linked immunosorbent assay (ELISA) utilizing two monoclonal antibodies directed against separate antigens of human serum PIICP, PIIANP, and PIIBNP. The kits for assay were provided by Shanghai Meilian and Beijing Biotopped Biological Technology Co. Ltd. These sandwich ELISAs were carried out according to the supplier’s protocols, and optical densities were determined using an automated reader (BioTek, USA). Intra- and inter-assay CVs were 9 and 15% for all the biomarkers, respectively.

### Statistical analysis

Statistical analysis was performed using the SPSS software, version 23.0. Age were expressed as mean ± standard deviation (SD). The normality test and the homogeneity test were performed. The two groups were compared using the *t* test (equal variance) or the *t*′ test (unequal variance) or the Wilcoxon rank sum test (non-normal distribution). Multiple sets of comparisons use variance analysis or Kruskal-Wallis *H* test (non-normal distribution), and the correlation between two factors uses Pearson or Spearman-related analysis. Differences were considered significant at *P* values of less than 0.05.

## Results

### Basic characteristics of the subjects

A total of 554 adults completed clinical examination, radiologic examination, and questionnaire survey. In matching the age and gender, 184 subjects were selected. Fifty-six cases were diagnosed as KBD (28 males and 28 females) and 63 individuals were included as internal control (28 males and 35 females) and 65 subjects were included as external control (27 males and 38 females). There were no statistically significant differences in age among the three groups (*F* = 1.565, *P* = 0.221). There were no statistically significant differences in sex among the three groups (*X*^2^ = 1.329, *P* = 0.514) (the detail was seen in Table 1).

**Table 1:** Basic characteristics of the subjects

	KBD	Internal control	External control
*N*	56	63	65
Male/female	28/28	28/35	27/38
Age	63.20 ± 6.18	61.48 ± 6.09	61.54 ± 5.63

### Correlations between serum levels of PIICP, PIIANP, and PIIBNP and age

**Fig. 1:**
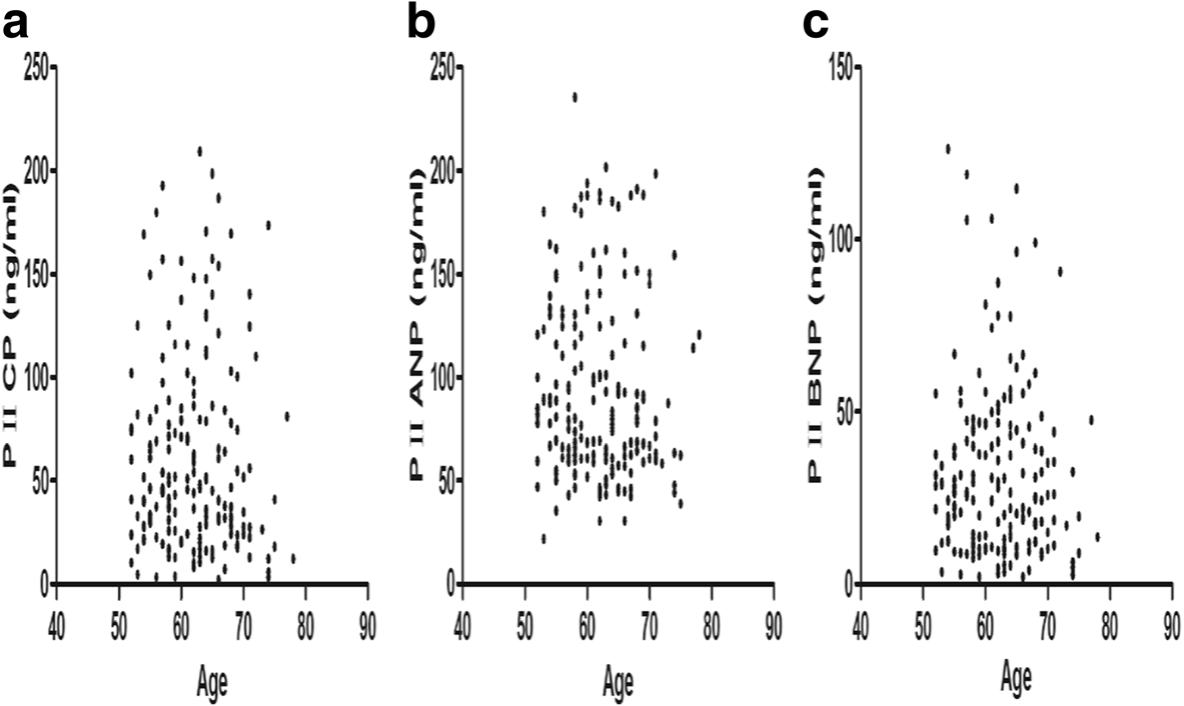
Correlations between the levels of PIICP, PIIANP, and PIIBNP in serum and age. **a** The correlations between serum levels of PIICP and age. **b** The correlations between serum levels of PIIANP and age. **c** The correlations between serum levels of PIIBNP and age

Scatter diagrams were used to show the correlation between the levels of PIICP, PIIANP, and PIIBNP in serum of the three groups and their age (Fig. 1). As shown in Fig. 1, there was no significant correlation between age and the levels of PIICP, PIIANP, and PIIBNP in serum.

In order to verify the accuracy of scatter diagrams, Spearman rank correlation was used to further analyze the correlation between the levels of PIICP, PIIANP, and PIIBNP in serum of the three groups and their age. The results from Spearman rank correlation analysis showed that rank correlation coefficient (*r*_s_) between the levels of PIICP, PIIANP, and PIIBNP and age was − 0.073 (*P* = 0.323), − 0.097 (*P* = 0.189), and − 0.052 (*P* = 0.486), respectively. And this result indicated that there was no linear correlation between age and the level of PIICP, PIIANP, and PIIBNP in serum.

### Comparison between serum levels of PIICP, PIIANP, and PIIBNP and sex

The Mann-Whitney *U* test and the Kruskal-Wallis *H* test were used to analyze the difference between serum levels of PIICP, PIIANP, and PIIBNP and sex. There were no statistically significant differences in serum levels of PIICP, PIIANP, and PIIBNP of different genders within the group (*P* > 0.05). However, there were statistical difference in serum levels of PIICP, PIIANP, and PIIBNP among different groups in both male and female (*H* = 9.83, *P* = 0.007; *H* = 14.74, *P* = 0.001; *H* = 9.71, *P* = 0.008; *H* = 12.87, *P* = 0.002; *H* = 11.39, *P* = 0.003; *H* = 14.00, *P* = 0.001) (Table 2). Besides, all these three indicators in the KBD group were obviously lower than those in the internal and external control groups.

**Table 2:** Relationship between the serum levels of the three biomarkers and sex

Biomarker (ng/ml)	Sex	KBD		Internal control		External control		*H*	*P*
		Median	P25,P75	Median	P25,P75	Median	P25,P75		
PIICP	Male	33.38	20.75,46.63	53.03	39.94,75.02	61.12	22.53,97.48	9.83	0.007
	Female	30.90	9.08,65.02	51.77	30.69,103.00	82.75	23.64,134.95	14.74	0.001
PIIANP	Male	65.61	56.79,91.42	98.85	74.09,152.67	80	57.12,160	9.71	0.008
	Female	65.37	48.60,89.36	92.98	67.57,151.66	80.40	67.66,114.52	12.87	0.002
PIIBNP	Male	17.15	11.29,24.19	30.63	21.89,40.54	26.82	11.99,47.41	11.39	0.003
	Female	11.61	4.79,32.39	25.90	19.43,47.78	41.24	12.06,54.89	14.00	0.001

### Comparison of the three biomarkers in serum in each group

The medians of serum PIICP, PIIANP, and PIIBNP levels in the KBD group were 32.43, 65.61, and 17.06 ng/ml, respectively; the medians of serum PIICP, PIIANP, and PIIBNP levels in the internal control were 51.86, 93.96, and 28.70 ng/ml, respectively; and the medians of serum PIICP, PIIANP, and PIIBNP levels in the external control were 67.04, 80.40, and 30.81 ng/ml, respectively. The serum PIICP, PIIANP, and PIIBNP levels were significantly different among the three groups (*H* = 23.198, *P* < 0.001; *H* = 23.937, *P* < 0.001; *H* = 23.999, *P* < 0.001).

**Fig. 2:**
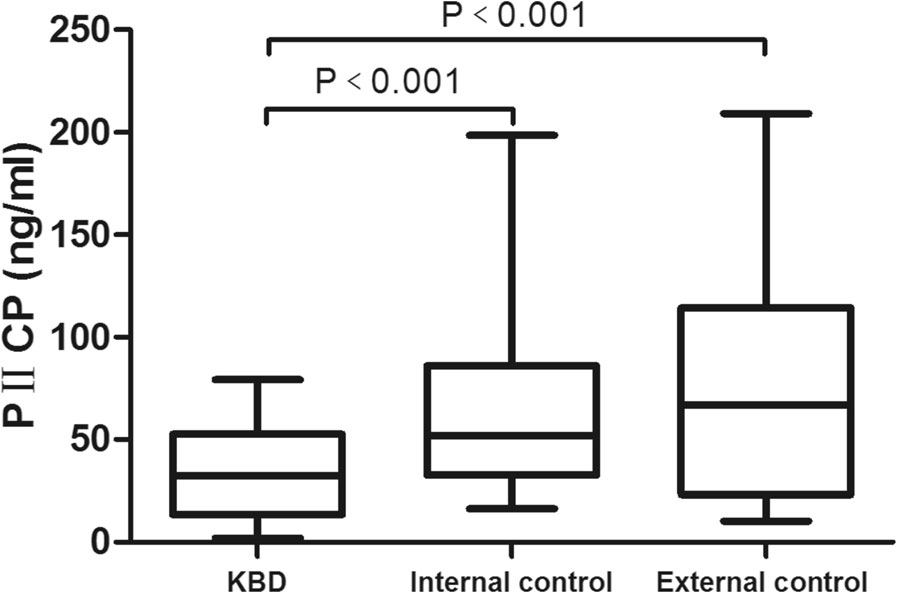
Serum level of PIICP in each group. PIICP levels in the KBD group were clearly lower than those in the internal and external control groups (*P* < 0.05)

**Fig. 3:**
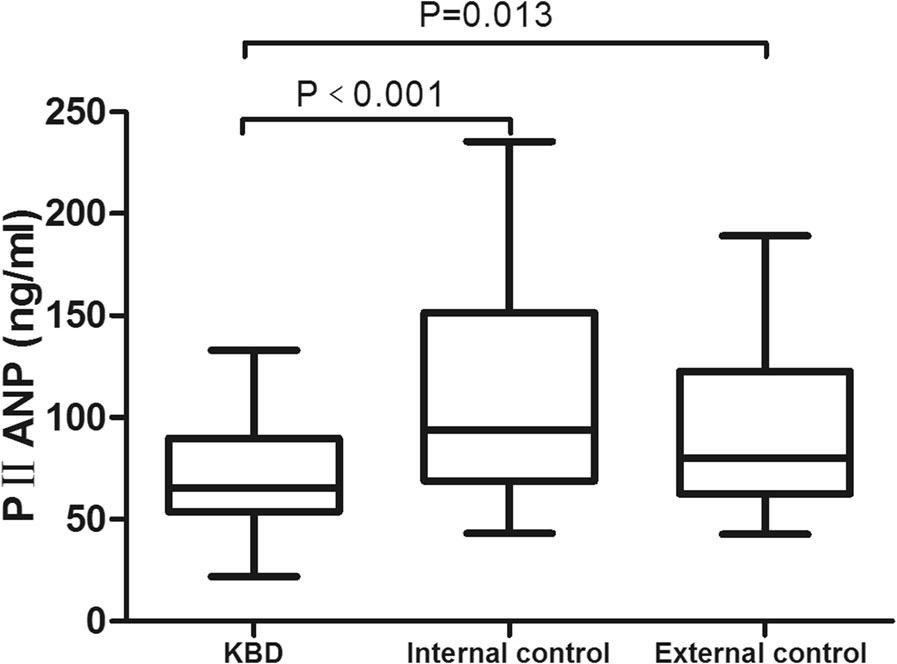
Serum level of PIIANP in each group. PIIANP levels in the KBD group were clearly lower than those in the internal and external control groups (*P* < 0.05)

**Fig. 4:**
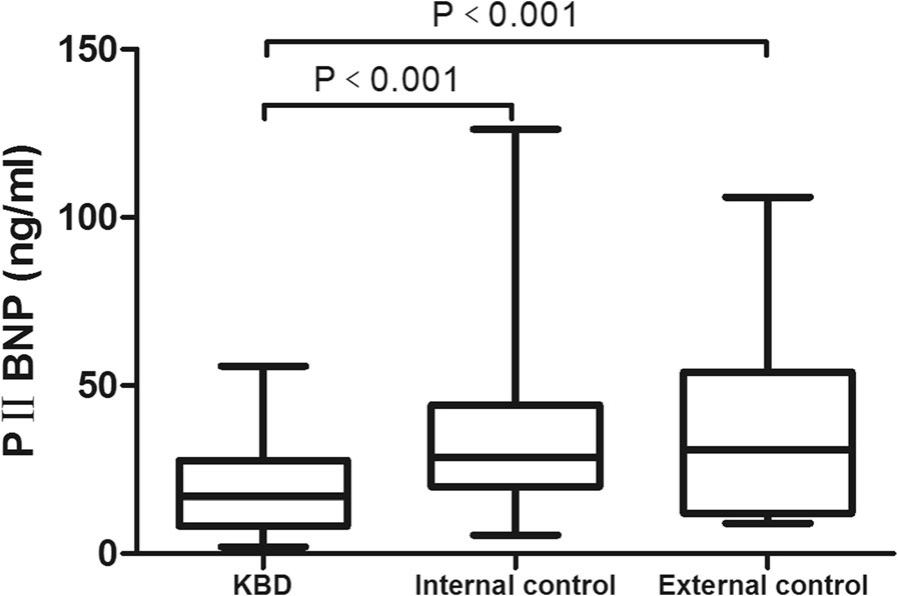
Serum level of PIIBNP in each group. PIIBNP levels in the KBD group were clearly lower than those in the internal and external control groups (*P* < 0.05)

Multiple comparisons using Dunnett’s T3 test revealed that serum levels of PIICP, PIIANP, and PIIBNP in the KBD group were clearly lower than those in the internal and external control groups (*P* < 0.05) (Figs. 2, 3, and 4). However, there was no significant difference between the internal control group and the external control group (*P* > 0.05).

## Discussion

KBD is an endemic, chronic, degenerative osteoarthropathy, which is characterized primarily by epiphyseal cartilage and articular cartilage deep chondrocyte necrosis and secondary hyperplasia and repair changes to pathological features, leading to osteochondral dysplasia, secondary degenerative joint disease [17]. So, the early stage of KBD is manifested as osteochondrosis, and then, these changes develop continuously, ultimately progresses to OA [18]. Therefore, the research on biomarker of OA is helpful for the research of KBD.

PIICP is the product of type II collagen synthesis; type II collagen is specifically expressed in cartilage tissues, so PIICP can specifically reflect cartilage metabolism and indicate the occurrence of OA. Sugiyama et al. conducted a 4-year follow-up of 172 early OA women with knee pain, and the data of 110 patients with successful follow-up showed that there was a mild positive correlation between body mass index and baseline PIICP level. At the same time, the degree of joint space stenosis was positively correlated with baseline PIICP level. Therefore, the level of PIICP in synovial fluid can predict the imaging progress of early knee OA [19]. Kobayashi et al. [20] detected the level of PIICP in synovial fluid of OA patients and found that the level of PIICP increased in early and middle stages of OA and decreased in the late stage of OA. The reason may be due to the damage of articular cartilage in the early stage of OA is not serious, but the compensatory increase of chondrocytes and enhancement of synthetic function are very obvious, so the level of PIICP elevates. In the late stage, the cartilage matrix is severely damaged, both the chondrocytes and the synthesis ability decreased, therefore the level of PIICP also decreased. In this study, we found that the serum levels of PIICP in KBD patients were significantly lower than those in internal and external control groups. The possible reason was that KBD mostly occurred in children and adolescents; with the growing of patients and the progress of disease, the cartilage matrix damaged more seriously and the synthesis ability decreased apparently. Therefore, serum concentrations of PIICP in adult KBD patients should be lower than those in normal subjects.

Type II collagen is synthesized as procollagen. During its maturation, there is a cleavage of C-terminal (PIICP) and N-terminal (PIINP) extension propeptides, which can serve as markers of cartilage synthesis. The N-terminal propeptide exist two variants (IIA/IIB), which arise from the alternative splicing of COL2A1 gene. These two procollagen forms differ from each other in the presence of exon 2 and in the distribution of their expression. Type IIA (PIIANP) contains exon 2 and is expressed by chondroprogenitor cells, whereas type IIB (PIIBNP) is devoid of exon 2 and is expressed in differentiated chondrocytes [21–23]. Although Sharif reported that serum PIIANP in knee OA progressors was higher than that in OA non-progressors [24], most of the previous studies have demonstrated that serum PIIANP decreased in knee OA patients compared to controls [25–30]. PIIBNP is believed to be the only procollagen expressed during the formation of type II collagen in healthy adult human cartilage [31]. Hayashi et al. found that PIIBNP can inhibit osteoclast survival and bone resorption via signal transduction through the αVβ3 integrins [32]. They proposed that PIIBNP may play a role in vivo in protecting cartilage from osteoclast invasion and also could be a new therapeutic strategy for decreasing bone loss. In this study, we observed that the serum levels of PIIANP and PIIBNP in KBD patients were significantly lower than those in the internal and external controls, suggesting a lack of type II collagen synthesis, resulting in deficiency of cartilage repair, further leading to the rapid development of KBD.

Some limitations are present in this study. Because in rural areas, men 40–60 years of age go out to work more, the survey population therefore included a relatively small number of healthy men. In addition, the mean age of patients with KBD is larger than previous studies. Meanwhile, we also needed to increase the number of patients to be verified in the follow-up experiment.

## Conclusions

In this study, the serum levels of PIICP, PIIANP, and PIIBNP in the KBD group were significantly lower than those in the internal and external control groups, but there was no significant difference between the internal control group and the external control group. The results preliminarily indicated that these three indicators could reflect the abnormal synthesis of type II collagen in KBD patients. And the results also suggested that these indicators might be used as potential biomarkers for the diagnosis of KBD.

## Abbreviations

BMI: Body mass index
KBD: Kashin-Beck disease
OA: Osteoarthritis
PIIANP: Type IIA procollagen N-terminal propeptide
PIIBNP: Type IIB procollagen N-terminal propeptide
PIICP: Type II procollagen C-terminal propeptide
